# Supplementary material for: Efficient single-photon emission via quantum-confined charge funneling to quantum dots
Source: Commun Mater. 2025 Nov 25;6(1):286. doi: 10.1038/s43246-025-01017-5 (PMC12727503; doi:10.1038/s43246-025-01017-5)
Supplement: Supplementary file 2 — Supplementary Information [file 43246_2025_1017_MOESM2_ESM.pdf]

Supplementary Information

Efficient single-photon emission via quantum-confined charge funneling to quantum dots

Sanghyeok Park[1,2], Khalifa M. Azizur-Rahman[1,2], Darryl Shima[3], Ganesh Balakrishnan[3], Jaeyeon Yu[1,2], Hyunseung Jung[1,2], Jasmine J. Mah[1,2], Samuel Prescott[4], Pingping Chen[2,5], Sadhvikas Addamane[1,2], Douglas Pete[1,2], Andrew Mounce[1,2], Ting Shan Luk[1,2], Prasad P Iyer[1,2], Igal Brener[1,2], Oleg Mitrofanov[4]

1. Sandia National Laboratories, Albuquerque, New Mexico 87185, United States
2. Center for Integrated Nanotechnologies, Sandia National Laboratories, Albuquerque, New Mexico 87185, United States
3. Center for High Technology Materials, University of New Mexico, Albuquerque, New Mexico 87106, United States
4. University College London, Electronic and Electrical Engineering, London WC1E 7JE, U.K.
5. Electrical, Computer and Energy Engineering, University of Colorado Boulder, Boulder, Colorado 80309, USA

Table of Contents:

Supplementary Note 1. ELM-QD sample design and MBE growth parameters

Supplementary Note 2. Identifications of QD location within the funnel

Supplementary Note 3. Photoluminescence signature of energy landscape modified quantum dots

Supplementary Note 4. Photoluminescence spectrum of ordinary quantum dots

Supplementary Note 5. Photoluminescence saturation

Supplementary Note 6. Overall efficiency of optically pumped single photon emitters

Supplementary Note 7. Second-order correlation function measurements

Supplementary Note 1. ELM-QD sample design and MBE growth parameters

ELM-QD sample design and MBE growth parameters		
**Material**	**Thickness**	**Description**
$Al_{0.4}Ga_{0.6}As$	70 nm	Capping layer
GaAs	1.7 nm	Quantum dot (grown using migration enhanced epitaxy)
-	-	Droplet etching
Al	0.6 ML	Droplet formation
$Al_{0.4}Ga_{0.6}As$	70 nm	Barrier (Ga droplets form and crystallize into disks in this layer)
GaAs	5 nm	GaAs interlayer
$Al_{0.75}Ga_{0.25}As$	500 nm	Sacrificial layer
GaAs	300 nm	Smoothing layer
GaAs (100)		Substrate

Supplementary Table 1. ELM-QD sample design and MBE growth parameters.

Supplementary Note 2. Identification of QD location within the funnel

QD themselves are not visible in the TEM images presented in Fig. 2 of the Article. We note that the QD dimeter is expected to be tens of nm and the probability of observing such a small object using cross-sectional TEM is small. However, Zhang. Y et al. (*Nano Lett.* 24, 10106-10113, 2024) showed that GaAs QDs fabricated by LDE have Al-rich side walls around the QD, which turns into Al-rich ‘puddles’ over 100 nm in diameter. The chance of observing these puddles with a TEM is much higher: they would appear as dark (Al-rich) streaks in TEM images (see Fig. 2a, Article), as illustrated in Suppl. Fig. 1. These streaks represent a thin layer with a higher Al concentration just above the interface, and we used them as indicators of the QD location. The streaks are seen only immediately above the interface, exactly where the QD are expected to form during the growth.

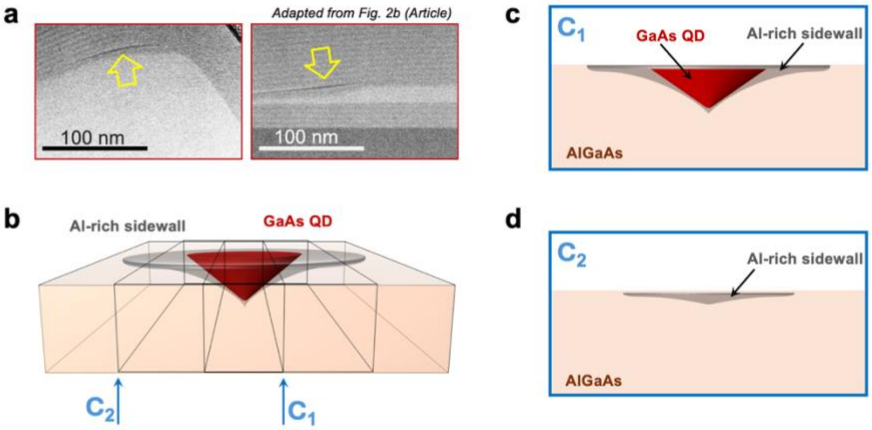

Supplementary Figure 1. Identification of ELM-QD location from TEM images. a. Two examples of TEM images showing dark streaks immediately above the AlGaAs funnel island. **b.** Schematic illustration of GaAs LDE QD structure containing the GaAs QD with an Al-rich sidewall. **c, d.** Schematic illustration of TEM observations for TEM cross-sections C_1 and C_2 shown in **b**. Depending on where the cross-sectional TEM is performed, the QD can appear as a triangular lighter feature with a darker outline as shown in C_1 (**c**), or as a dark steak as shown in C_2 (**d**).

Supplementary Note 3. Photoluminescence signature of energy landscape modified quantum dots

The charge carrier funnels modify the energy landscape around the QDs, attracting charge carriers and increasing the excitation (and the overall) efficiency of the QDs. As a result, light emission from these energy landscape modified quantum dots (ELM-QDs) is visibly stronger (over one order of magnitude), as discussed in the Article (see Fig. 1d, Fig. 4b). In addition, the photoluminescence (PL) spectrum from the region around these QDs carries a signature of the energy landscape, with significant PL emission from the area immediately surrounding the QDs, i.e. from the funnel itself. Using spectrally-filtered PL imaging, we can selectively suppress the emission from either the funnel or the ELM-QDs to visualize them.

Supplementary Figure 2 shows PL images of a 40 x 40 μm^2 area near one of the ELM-QDs using three different optical filters placed before an imaging camera. Without any filter, a PL image (not shown) displays one strongly emitting QD surrounded by ordinary QDs. When a short-wavelength band pass filter is introduced, the PL from the QD itself is suppressed, and a 5-7 μm diameter halo becomes visible around the QD (Suppl. Fig. 1a, *top row*). The halo is caused by emission of photons with energies higher than the QD-emitted photon, and it comes from interband recombination of charge carriers within the funnel. If the bandpass filter is replaced with a longpass filter suppressing the funnel emission, we expect to observe only the QDs. However, the strong emission from the QD saturates the image making it difficult to differentiate between the funnel and the QD (Suppl. Fig. 1a, *middle row*). When the transmission edge of the bandpass filter is shifted further to the longer wavelengths, and we can finally observe localized PL from only the ELM-QD itself (*bottom row*).

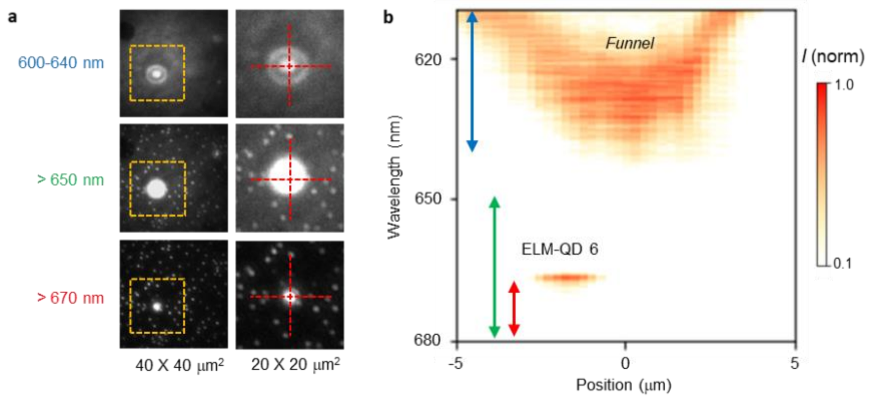

Supplementary Figure 2. a. PL images of ELM-QD and ordinary QDs obtained with different spectral filters: bandpass filter: 600-640 nm (top row); longpass filter: 650 nm (middle row); and longpass filter: 670 nm (bottom row), with the transmission bands indicated in the PL map. The right column images show magnified images of the area in the dashed box in the left column. **b.** Spatial distribution of PL along a line scan across the ELM-QD in (**a**). The colored arrows indicate the spectral transmission windows for the filters used in (**a**).

These PL images are consistent with the confocal PL map displayed in Suppl. Fig. 2b. They are also consistent with the charge carrier funnel structure determined by TEM (see Article, Fig. 2) and with the bandgap energy calculations shown in Fig. 2e of the Article. The PL halo feature can be used to locate ELM-QDs within the wafer.

We note that the energy landscape in the funnels varies, and it can be characterized using the confocal PL maps (see Suppl. Fig. 2b and Fig. 3 in the Article). In Suppl. Fig. 3 we show PL maps for three additional ELM-QDs (ELM-QD 6, 7 and 8). All the maps show the common inverted bell-shaped PL signature.

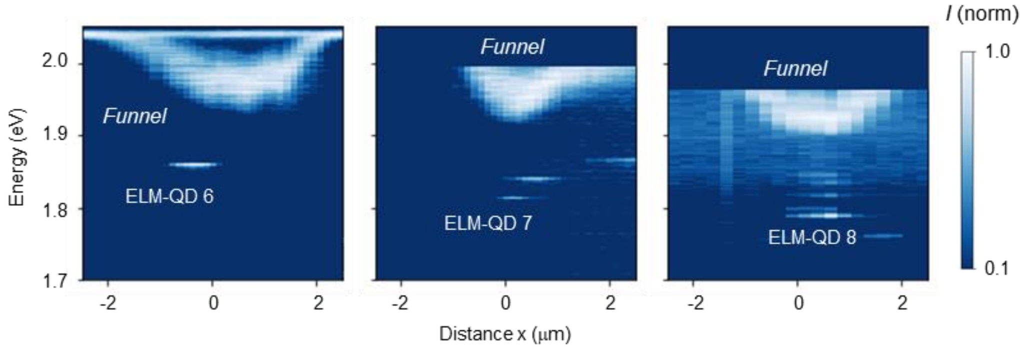

Supplementary Figure 3. Spatial distribution of PL along a line scan across ELM-QD 6, 7 and 8.

Supplementary Note 4. Photoluminescence spectrum of ordinary quantum dots

PL spectra of ordinary LDE GaAs QDs (o-QDs) were compared with the spectra of ELM-QDs (presented in the Article in Figs. 3 and 4). Supplementary Figure S4 shows an example of the o-QD spectrum; it was obtained with a micro-PL setup using a 50X microscope objective lens and the pump power of 5.6 nW.

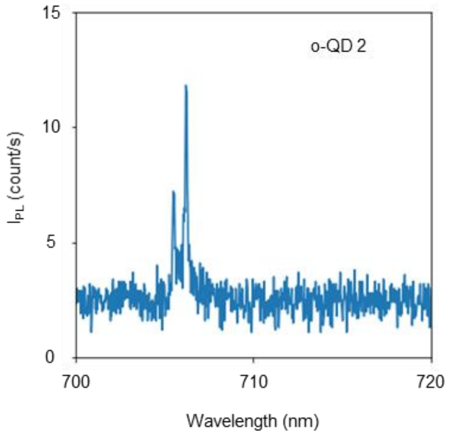

Supplementary Figure 4. PL spectrum of an ordinary GaAs QD (o-QD 2) excited with the incident power of 5.6 nW (excitation laser wavelength λ = 515 nm).

Supplementary Note 5. Photoluminescence saturation

The increase in excitation efficiency for the ELM-QDs was observed in PL saturation curves measured for the ELM-QDs and o-QDs. Here, we compare PL intensity as a function of the QD excitation power for ELM-QD 12 and o-QDs (see Supplementary Note 4). ELM-QD 12 shows a linear increase in PL intensity for the pump power below ~50 nW exhibiting saturation at ~100 nW (Suppl. Fig. 4). In contrast, PL intensity for the ordinary QDs continues increasing with the pump power, albeit slightly sub-linearly. Most importantly, the ELM-QD efficiency is noticeably higher: at pump powers below 100 nW, it is ~8-10 times higher for ELM-QD 12 compared to the o-QDs.

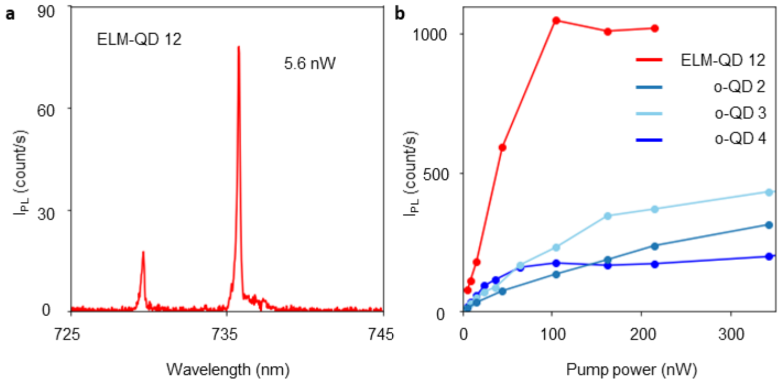

Supplementary Figure 5. a. PL spectrum of ELM-QD 12 (excitation power of 5.6 nW). **b.** PL peak intensity as a function of pump power for ELM-QD 12 (red line) and for ordinary QDs (o-QD 2-4, blue lines).

Below the saturation level (~100 nW), the main exciton peak dominates the spectra of ELM-QDs. At ~100 nW, ELM-QD 1 starts showing several emission lines corresponding to exciton complexes. Supplementary Figure 6 displays the spectrum of ELM-QD1 for the excitation power of 100, 270 and 430 nW. As the pump power increases, the main exciton peak diminishes, and the emission from exciton complexes becomes more pronounced. This occurs because a higher charge carrier density facilitates the transition of excitons into exciton complex states.[1-4]

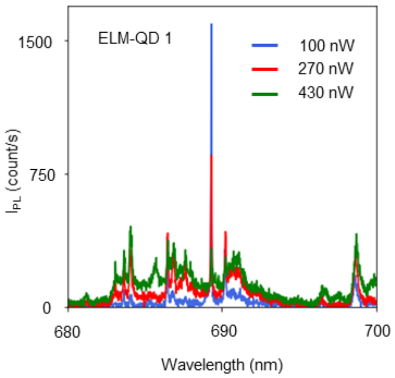

Supplementary Figure 6. PL spectra of ELM-QD1 for the optical pump power of 100 nW, 270 nW, and 430 nW.

Supplementary Note 6. Overall efficiency of optically pumped single photon emitters

To compare the efficiency of ELM-QD emitters with other reported SPEs, we evaluated their overall quantum efficiency. For clarity, we define the overall quantum efficiency η as the number of single photons generated per one pump photon. The overall efficiency is a product of the internal quantum efficiency η_{int}, the excitation efficiency η_{x} and the outcoupling efficiency η_{oc}:

$$\eta = \eta_{\text{int}}\eta_{\text{x}}\eta_{\text{oc}}.$$

Efficiency of ELM-QDs

Evaluation of excitation efficiency for ELM-QDs	
	**EML-QD**
Detection Efficiency[1]	0.008
Excitation Power (nW)[2]	100
Excitation wavelength (nm)	520
# of Exc. Photons (s^{-1})	2.6×10^{11}
Experimental Count Rate (Hz)[3]	1×10^{4}
Photon Emission Rate[4]	1.25×10^{6}
Overall Quant. Efficiency	4.8×10^{-6}
Outcoupling Efficiency[5]	0.005
Excitation Efficiency	1×10^{-3}
Excitation Method	Non-resonant

[1] Detection Efficiency is defined as the overall optical detection efficiency, which includes the efficiency of the optical setup (50%), fiber coupler efficiency (15%), long-pass and short-pass filter efficiency (25%), and SNSPD detection efficiency at 700nm (85%).
[2] Excitation power
[3] Experimental Count Rate is the measured photon count rate
[4] Photon emission rate is the number of emitted photons from the quantum dot per second which can be estimated as the experimental count rate divided by the detection efficiency.
[5] Outcoupling Efficiency is estimated using numerical (FDTD) simulations

Supplementary Table 2. Summary of experimental results and extracted values of overall quantum efficiency, outcoupling efficiency and excitation efficiency of ELM-QDs.

For reference, Supplementary Table 3 gives a summary of experimental results from recently published articles on QD-based SPEs, and extracted values of the overall quantum efficiency, outcoupling efficiency and excitation efficiency (Ref. 18, 19, 56 and 20 in the Article):[5, 10, 14, 15]

Evaluation of excitation efficiency				
	**Ref.18 (10)**	**Ref. 56 (5)**	**Ref. 19 (14)**	**Ref. 20 (15)**
Detection Efficiency[1]	0.017	0.0405	0.69	0.71
Excitation Power (nW)[2]	2000	1000	25	10.24
Excitation wavelength (nm)	860	800	922	844
# of Exc. Photons (s^{-1})	8.7×10^{12}	4.0×10^{12}	1.2×10^{11}	4.3×10^{10}
Experimental Count Rate (Hz)[3]	0.7×10^{6}	8.2×10^{5}	4.0×10^{7}	1.4×10^{7}
Photon Emission Rate[4]	4.0×10^{7}	2.0×10^{7}	5.8×10^{7}	2.0×10^{7}
Overall Quant. Efficiency	4.6×10^{-6}	5.0×10^{-6}	4.8×10^{-4}	4.7×10^{-4}
Outcoupling Efficiency	0.8	0.2544	0.96	0.939
Excitation Efficiency	5.8×10^{-6}	2.0×10^{-5}	5.0×10^{-4}	5.0×10^{-4}
Excitation Method	Non-resonant	Non-resonant	Resonant	Resonant

[1] Detection Efficiency is defined as the overall optical detection efficiency, which considers all the losses in the optical setup, i.e. beam splitters, fiber couplers, filters, etc.
[2] Excitation power or Saturation power
[3] Experimental Count Rate is the measured photon count rate provided in the references.
[4] Photon emission rate is the number of emitted photons from the quantum dot per second which can be estimated as the experimental count rate divided by the detection efficiency.

Supplementary Table 3. Summary of experimental results from recently published articles on QD-based SPEs (Article Refs. 18, 19, 20 and 56),[5,10,14,15] and extracted values of the overall quantum efficiency, excitation efficiency and outcoupling efficiency.

For example, for non-resonant excitation in Ref. 10 (Article Ref. 18),[10] the photon emission rate is ~4×10^7 (s^{-1}), while the flux of excitation photons is ~8.7×10^{12} (s^{-1}) (calculated from the pump power and the excitation wavelength). Therefore, the overall quantum efficiency is ~4.6×10^{-6}. The outcoupling efficiency η_{oc}: (the efficiency of coupling into the first lens) was 80 %, and therefore the excitation efficiency is 5.8×10^{-6}.

Under resonant excitation, the excitation efficiency can exceed 10^{-4}. For example, in Ref. 14 (Article Ref. 19),[14] the photon emission rate is 5.8×10^7 (s^{-1}), while the number of input photons is 1.2×10^{11} (s^{-1}). The overall efficiency therefore is 4.8×10^{-4}. With the reported outcoupling efficiency of 96 %, the excitation efficiency is 5.0×10^{-4}.

Estimation of outcoupling efficiency for ELM-QDs

We estimated the outcoupling efficiency using electromagnetic simulations. The sample structure used for the simulations is shown in Suppl. Fig. 7a. The QD was modeled as an in-plane polarized dipole emitter, embedded at the center of a 140-nm-thick $Al_{0.4}Ga_{0.6}As$ layer on top of a 500-nm-thick $Al_{0.75}Ga_{0.25}As$ layer and a GaAs substrate. The outcoupling efficiency is defined as the ratio of the emitted power into free space (within the collection angle corresponding to a NA of 0.4) to the total emission power from the dipole.

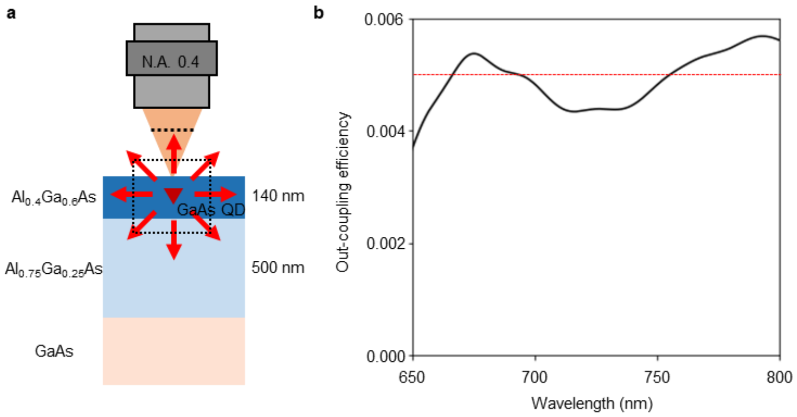

Supplementary Figure 7. a. Schematic of the simulated structure with embedded point source for outcoupling efficiency estimations. **b.** Simulated normalized outcoupling efficiency spectrum for NA 0.4 (defined as the ratio of the emitted power into the lens to the total emitted power). The red line indicates the level of 0.005.

The outcoupling efficiency for ELM-QDs embedded in the high index material is low (~0.005) due to photon trapping in the substrate. However, the efficiency can be improved significantly by engineering photonic properties of the surface or/and by introducing a back mirror.

Estimation of excitation efficiency

The excitation efficiency η_x can be estimated from the outcoupling efficiency η_{oc} and the overall quantum efficiency η as $\eta_x \cong \eta/\eta_{oc}$ (assuming that the internal quantum efficiency $\eta_{int} \cong 1$). The overall efficiency for ELM-QDs is ~5×10^{-6} and the excitation efficiency is estimated to be ~0.1%. We note that this value exceeds the excitation efficiency for the reported QDs (Suppl. Fig. 8b), even for QDs embedded in specially designed photonic structures, for both the non-resonant and resonant excitation schemes. The overall quantum efficiency is comparable to that observed in QDs with specially designed outcoupling solutions (Suppl. Fig. 8a). [5-13]

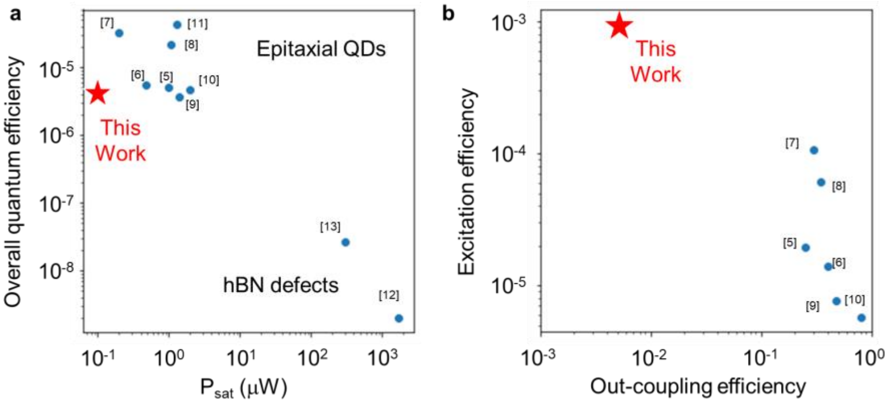

Supplementary Figure 8. a. Comparision of our ELM-QDs with other reported quantum emitters in terms of the saturation pump power and overall quantum efficiency. **b.** Comparision of our ELM-QDs with other reported epitaxially grown QDs in terms of outcoupling efficiency and excitation efficiency.

Estimation of brightness

The outcoupling efficiency allows us to estimate the QD brightness, defined as $B = \beta\eta_{lens}p_x\eta_{QE}$, where $\beta\eta_{lens}$ is the outcoupling efficiency, p_x is the occupation factor of the QD state, and the η_{QE} is the internal quantum efficiency of the QD. For our ELM-QDs, the outcoupling efficiency into NA = 0.4 is ~0.005 (Suppl. Fig. 7). Assuming a pump power higher than the saturation power, the occupation factor, p_x is proportional to $1 - \exp\left(-\frac{P}{P_{sat}}\right)$, and it could approach unity. Internal quantum efficiency for GaAs QD can also be assumed close to unity. Under such high-power excitation conditions, the brightness of ELM-QDs is estimated to be 0.005.

Supplementary Note 7. Second-order correlation function measurements

The ELM-QDs show the behavior of single photon emitter in the second-order correlation measurements. To isolate the main QD emission peak spectrally, we used a pair of filters (FEL0700, Thorlabs and BrightLine® single-band bandpass filter (692/40 nm) from IDEX health & Science). By adjusting the angle of incidence for the filters, we obtained a 2.1 nm wide passband at ~689 nm for ELM-QD 1 (as shown in Suppl. Fig 9a). However, the passband was still too wide for the QD peak, and it could not fully block emission at other wavelengths, creating a background signal in our second-order correlation measurements.

The second-order correlation measurements were conducted in a time-tagged mode using PicoHarp 300 (PicoQuant). The time window and time bin for the autocorrelation calculation were set as 16 ns and 16 ps. The measured $g^{(2)}$ curve was normalized to the mean value of measured autocorrelation in the time window.

To eliminate the background signal, we used the random coincidence correlation correction.[16] We fitted the main peak in the PL spectrum with a Lorentzian function (red dashed line in Suppl. Fig. 9b) with the center energy of 1.7991 eV and the linewidth of 0.042 meV (4.2×10^{-5} eV). It allowed us to estimate that within the filtered spectral range (orange shaded regions in Suppl. Fig. 9) the PL contribution from the main exciton peak was 0.78 of the total photon count. The random coincidence correlation corrected result for $g^{(2)}$ was presented in Fig. 4c in the Article. For completeness, we also show the second order correlation measurement result without applying the correction in Suppl. Fig. 9c. Even without the correction, the ELM-QD meets the standard for the single photon source with $g^{(2)}(0) = 0.33$.

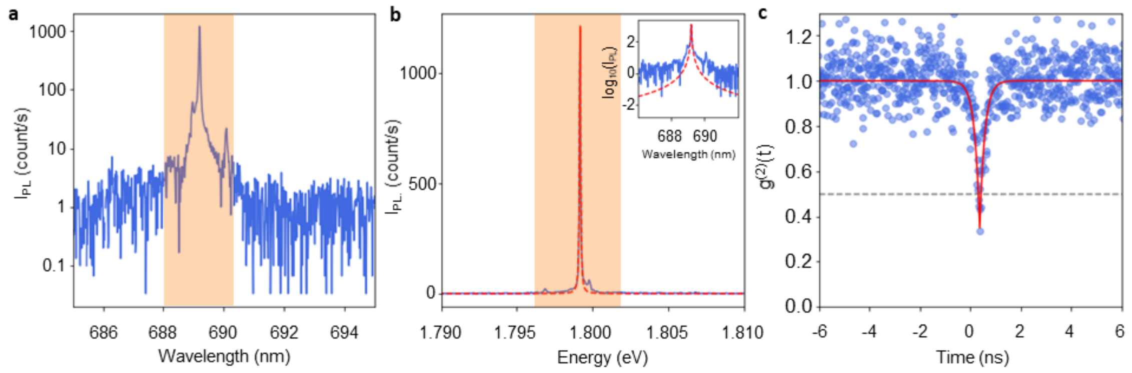

Supplementary Figure 9. a. Spectrally filtered PL of ELM-QD 1 for the pump power of 50 nW (logarithemic scale). The shaded region shows the 2.1 nm spectral window used for PL emission filtering. **b.** Spectrally filtered PL of ELM-QD 1 (linear scale) with Lorentzian fitting (red dashed lines). **c.** Second-order correlation function for ELM-QD 1 without the random coincidence correlation correction.

References

1. Juska, G., et al. Towards quantum-dot arrays of entangled photon emitters. *Nature Photon* 7, 527–531 (2013).

2. Zieliński, M.et al. Excitonic complexes in natural InAs/GaAs quantum dots. *Phys. Rev. B*. 91,085303 (2015).

3. Abbarchi, M. et al. Energy renormalization of exciton complexes in GaAs quantum dots. *Phys. Rev. B*, 82, 201301 (2010).

4. Yu, Y. et al. Fundamental limits of exciton-exciton annihilation for light emission in transition metal dichalcogenide monolayers. *Phys. Rev. B*. 93, 201111 (2016).

5. Li, X., Liu, S., Wei, Y. et al. Bright semiconductor single-photon sources pumped by heterogeneously integrated micropillar lasers with electrical injections. *Light Sci Appl* 12, 65 (2023).

6. Larocque, H., Buyukkaya, M.A., Errando-Herranz, C. et al. Tunable quantum emitters on large-scale foundry silicon photonics. *Nat Commun* 15, 5781 (2024).

7. Reimer, M., Bulgarini, G., Akopian, N. et al. Bright single-photon sources in bottom-up tailored nanowires. *Nat Commun* 3, 737 (2012).

8. Claudon, J., Bleuse, J., Malik, N. et al. A highly efficient single-photon source based on a quantum dot in a photonic nanowire. *Nature Photon* 4, 174–177 (2010).

9. Sapienza, L., Davanço, M., Badolato, A. et al. Nanoscale optical positioning of single quantum dots for bright and pure single-photon emission. *Nat Commun* 6, 7833 (2015).

10. Gazzano, O., Michaelis de Vasconcellos, S., Arnold, C. et al. Bright solid-state sources of indistinguishable single photons. *Nat Commun* 4, 1425 (2013)

11. Schlehahn, A, et al. Generating single photons at gigahertz modulation-speed using electrically controlled quantum dot microlenses. *Appl. Phys. Lett.* 108, 021104 (2016)

12. Gan, L, et al. Large-Scale, High-Yield Laser Fabrication of Bright and Pure Single-Photon Emitters at Room Temperature in Hexagonal Boron Nitride. *ACS Nano* 16, 9, 14254-14261 (2022)

13. Wang, X.-J., et al. Enhanced brightness of quantum emitters via in situ coupling to the dielectric microsphere. *Appl. Phys. Lett.* 123, 133106 (2023)

14. Tomm, N., *et al.* A bright and fast source of coherent single photons. *Nat. Nanotechnol.* **16**, 399–403 (2021).

15. Ding, X., et al. High-efficiency single-photon source above the loss-tolerant threshold for efficient linear optical quantum computing. *Nat. Photon.* 19, 387-391 (2025)

16. Brouri. R. et al., Photon antibunching in the fluorescence of individual color centers in diamond. *Opt. Lett.* 25, 1294 (2000)
